# Auditory affective content facilitates time-to-contact estimation of visual affective targets

**DOI:** 10.3389/fpsyg.2023.1105824

**Published:** 2023-05-03

**Authors:** Feifei Lu, You Li, Jiajia Yang, Aijun Wang, Ming Zhang

**Affiliations:** ^1^Department of Psychology, Research Center for Psychology and Behavioral Sciences, Soochow University, Suzhou, China; ^2^College of Chinese Language and Culture, Jinan University, Guangzhou, China; ^3^Applied Brain Science Lab, Faculty of Interdisciplinary Science and Engineering in Health Systems, Okayama University, Okayama, Japan; ^4^Cognitive Neuroscience Laboratory, Graduate School of Interdisciplinary Science and Engineering in Health Systems, Okayama University, Okayama, Japan

**Keywords:** time-to-contact (TTC) estimation, threat, audiovisual integration, velocity, presentation time

## Abstract

Reacting to a moving object requires an ability to estimate when a moving object reaches its destination, also referred to as the time-to-contact (TTC) estimation. Although the TTC estimation of threatening visually moving objects is known to be underestimated, the effect of the affective content of auditory information on visual TTC estimation remains unclear. We manipulated the velocity and presentation time to investigate the TTC of a threat or non-threat target with the addition of auditory information. In the task, a visual or an audiovisual target moved from right to left and disappeared behind an occluder. Participants’ task was to estimate the TTC of the target, they needed to press a button when they thought that the target contacted a destination behind the occluder. Behaviorally, the additional auditory affective content facilitated TTC estimation; velocity was a more critical factor than presentation time in determining the audiovisual threat facilitation effect. Overall, the results indicate that exposure to auditory affective content can influence TTC estimation and that the effect of velocity on TTC estimation will provide more information than presentation time.

## 1. Introduction

To coordinate movement and events in a dynamic environment, the brain must predict when those events will occur, and the ability of temporal prediction is important for daily survival. To avoid potentially dangerous objects, one must use time or spatial information to estimate the moment it reaches a position to avoid threatening objects. The time it takes for threatening objects to reach one position or a specific position is the time-to-collision or time-to-contact (TTC) (DeLucia and Liddell, 1998; DeLucia et al., 2003). Estimation of TTC reflects the comprehensive processing ability of spatiotemporal information (Chang and Jazayeri, 2018; Patrick and Anderson, 2021). TTC estimation (TTCE) enables individuals to better estimate the actual remaining time of object collision in a complex and changeable environment, and its accuracy directly affects individuals’ adaptation to the environment and performance of various perception-motor tasks.

Recent studies have shown that TTC estimation is affected by the affective content of stimuli: threatening images of frontal attacks, facial expressions, or threatening animals (Brendel et al., 2012; Vagnoni et al., 2012, 2015, 2017; DeLucia et al., 2014). Compared with neutral and happy faces, Brendel et al. (2012) found that people often underestimate the TTC of an angry face, so it is reasonable to expect that the “threat advantage” effect is a factor in TTC estimation, albeit the effect was small. However, DeLucia et al. (2014) compiled a set of facial expressions and found that TTC estimation of neutral and friendly facial expressions was overestimated, whereas threatening facial expressions were overestimated by a smaller magnitude. This finding indicated no significant differences between TTC estimation of threatening and non-threatening facial expressions. Although the results are controversial, they suggested that the affective content of facial expressions may affect participants’ TTC estimations. We expected that not only visual threats, but also stimuli from auditory modalities can influence TTC.

Many studies have shown that auditory stimuli affect visual events (Braly et al., 2021; Patrick and Anderson, 2021), because the auditory domain has many advantages over the visual system; specifically, sound presentation serves as an alert or provides warning content that leads to a high arousal state (Juslin and Västfjäll, 2008; Asaoka and Gyoba, 2016). For example, Braly et al. (2021) found that a snake sound was judged as arriving earlier than the corresponding control sound, providing partial support that threat-related effects on TTC estimates for visual objects can also occur in the auditory domain. Chotsrisuparat et al. (2017) found that auditory rhythms affect the expected reappearance of an occluded moving object. This means that the auditory system can complement the sensory system by providing information on the events occurring outside one’s visual field (Ferri et al., 2015).

However, for the effect of threat content on TTC, at least two major questions have not been answered. First, a question that has been neglected is whether TTC estimation of threatening visual stimuli is modulated by accompanied auditory affective cues. It is reasonable to expect that observers may use both auditory and visual information to better predict an object’s future position rather than relying only on unimodal information (Prime and Harris, 2010). As mentioned, TTC estimation can be influenced by auditory stimuli (Neuhoff et al., 2014; Chotsrisuparat et al., 2017; King and Prime, 2019), and threatening auditory stimuli and visual facial expressions can distort (lengthen or shorten) subjective duration (Droit-Volet et al., 2004; Gil et al., 2007; Gil and Droit-Volet, 2011; Fallow and Voyer, 2013; Wackerman et al., 2014; Voyer and Reuangrith, 2015; Droit-Volet and Gil, 2016; Corke et al., 2018).

Second, we aim to examine whether there is a “central-tendency effect” in TTC estimation meaning that the TTCs with higher velocities are overestimated whereas the TTCs with medium velocities are estimated more precisely (Makin et al., 2008; Bennett et al., 2010; Li et al., 2015; Fath et al., 2018; Tian et al., 2018a). Tian et al. (2018a) found that with increasing velocity, the value of TTC overestimation increased. Li et al. (2015) explored the effect of velocity on TTC through fMRI studies and found that the behavioral velocity-induced time dilation effect and the connectivity between the early visual cortex and TTC network increased with increasing velocity.

Furthermore, whether the velocity and presentation time affect the threatening effect of TTC estimation remains unclear. Many studies have confused target velocity with the presentation time of the target before it is intercepted (Mason and Carnahan, 1999). In addition to top-down factors, bottom-up kinematic information (e.g., velocity and distance) and temporal cues affect TTC estimation (Brenner and Smeets, 2015; Chang and Jazayeri, 2018; Battaglini and Ghiani, 2021). Previous studies found that the target presentation time was positively correlated with the TTC estimation response of the participants (Peterken et al., 1991). This means that the longer the stimulus presentation time, the better the accuracy of the TTC estimation. In general, a longer presentation time improves response accuracy, possibly because the time interval for extracting the target motion information is long (Peterken et al., 1991; Mason and Carnahan, 1999). At a lower velocity, participants may have enough time to extract the relevant information for the predicted moving objects. However, a higher velocity with a longer presentation time could not improve the response accuracy because the reaction time was limited, which made the judgment task more challenging.

The present study aimed to investigate the effect of additional auditory information on TTC estimation by controlling the velocity and presentation time. We used a single target that moved in a horizontal direction and asked participants to press a button at the exact time at which they determined that the moving threatening (or non-threatening) target would have reached a marked location on the occlude (a red bar). In Experiment 1, participants estimated the TTC of a visual or an audiovisual target moving at different velocities. To preclude the “central-tendency effect,” we conducted Experiment 2 with the grouped velocity condition. We hypothesized that TTC estimation for a threatening target would be more accurate than for a non-threatening target, and such a facilitation effect would be stronger with the addition of an auditory affective content condition than in the visual condition. In Experiment 3, we investigated the role of velocity and presentation time in TTC estimation. We hypothesized that velocity would play a more important role than presentation time.

## 2. Experiment 1: auditory affective content affects different velocities of TTC estimation

### 2.1. Method

#### 2.1.1. Participants

Thirty-four participants (mean age 21.98 ± 1.8 years, range 19–26, 25 females) participated in Experiment 1. All participants were right-handed and had normal or corrected-to-normal vision and normal color vision, and none of them had a history of neurological or psychiatric disorders, as self-reported. Informed consent was obtained from each participant before the experiment was conducted according to the Declaration of Helsinki before their inclusion. All the participants were naive to the purpose of the experiment. The study was approved by the Academic Committee of the Department of Psychology, Soochow University, China.

To evaluate the statistical testing power in the present study, sensitivity analysis was conducted on the two-side paired *t* test through the software G*Power 3.1 (Faul et al., 2007). The input parameters were as follows: *α* err prob = 0.05, power (1-*β* err prob) = 0.80, and total sample size = 34. The output parameters of Cohen’s *d* = 0.50 were calculated, which reached a medium effect size.

#### 2.1.2. Apparatus, stimuli, and experimental setup

The visual stimuli were 60 color photographs from the “NimStim Set of Facial Expressions” (Tottenham et al., 2009), including 15 from each of the four categories of the open-mouth version (i.e., male-angry, female-angry, male-happy, female-happy). The photographs were 200 × 200 pixels. The backgrounds of the photographs were replaced with a homogenous gray color (RGB: 213, 213, 213). Auditory stimuli consisted of 60 voices including 15 from each of the four categories (i.e., male-threatening, female-threatening, male-non-threatening, female-non-threatening) with a screamed or a happy intonation containing no meaningful syllables. Voices were cropped and resized using Adobe Audition CC with a sampling rate of 44.1 kHz, lasting for 1,500 ms (Adobe Systems, San Jose, CA). Please refer to online resources for details.

Stimuli were generated with MATLAB (The MathWorks) with the Psychophysics Toolbox-3 (Pelli and Vision, 1997) and were displayed on a View Sonic P225f VS10284 monitor with a refresh rate of 60 Hz. The auditory information in the experiment was presented through a headset (HD200 PRO) at 60–70 dB. The screen resolution was 1920 × 1,440 pixels. To ensure that participants could perceive the sound stimulus moving with the picture stimulus, we processed the sound stimulus with MATLAB, and the dB amplitude changes of the left channel increased from small to large but dB amplitude changes of the right channel decreased from large to small. At the same time, to ensure that the participants perceived the sound stimulus as changing with velocity, we also varied the ratio of the sound correspond to different velocities. The experiment was carried out one participant at a time in a dim soundproof room. The participants were seated in a dark room, 57 cm from the display screen, and rested their heads on a chin bracket to keep their eyes level with the central position of the display screen.

### 2.2. Experimental procedures and design

**Figure 1:**
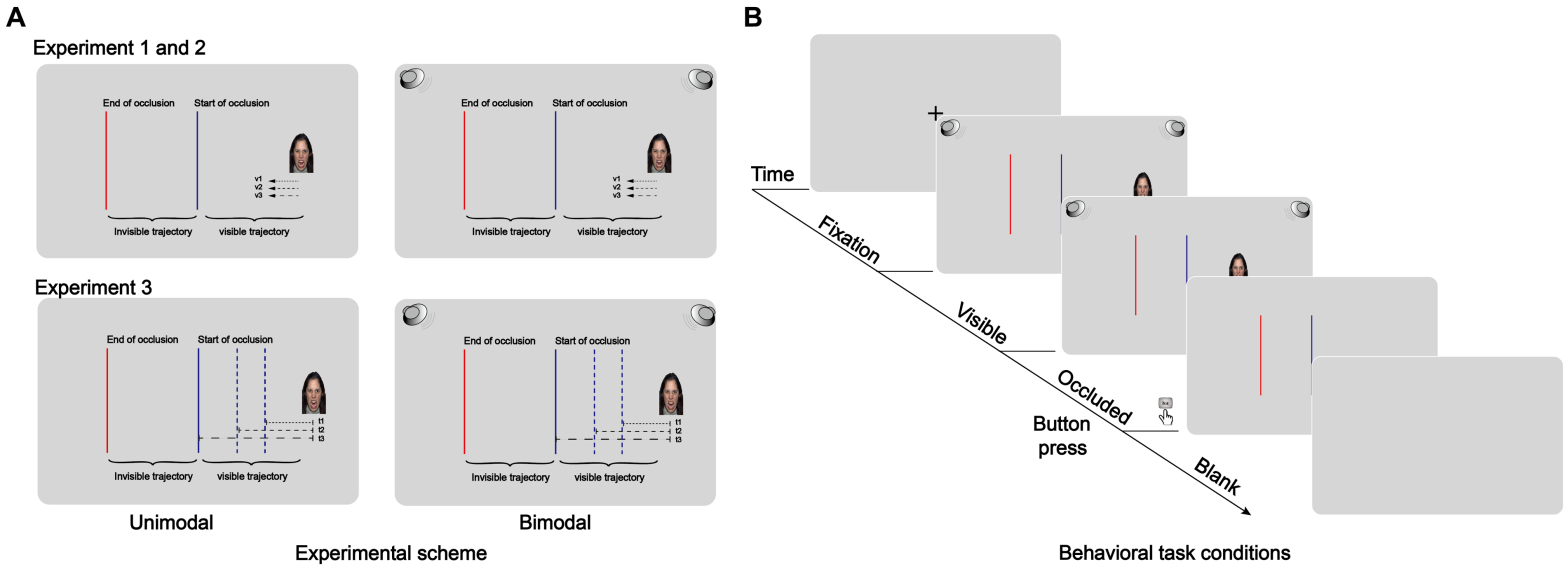
**(A)** Behavioral task conditions. Schematic illustration of Experiment 1, Experiment 2, and Experiment 3 in which a target moved along a path that was divided into two segments: one was a visible trajectory extending from an initial point to a blue bar that indicated the start of occlusion, and the other was an invisible trajectory extending from a blue bar to a red bar that indicated the end of occlusion. The target velocity or presentation time of the time-to-contact (TTC) was randomly varied. **(B)** General schematic representation of the TTC. The intertrial interval was 500 ms. Bimodal moving targets were a facial expression paired with a corresponding voice. The target was presented at a constant velocity before reaching the blue line and disappearing. Participants had to estimate the TTC of this target with the second red line by pressing the “space” key after it disappeared. The visual-only conditions were identical to the bimodal conditions except that the visual conditions were presented independently.

Each trial began with a 500-ms fixation period followed by the presentation of the target. The target began on the right side of the screen and moved leftward for 1,500 ms until it reached the beginning point of an occluder and then disappeared. The beginning point (right edge) of the occluder was a thin vertical blue line, 100 pixels from the screen center on the left. The participants were told that although it disappeared, the target would continue moving behind the occluder until it contacted the destination of the occluder. The destination (left edge) of the occluder was a thin vertical red line 500 pixels from the left of the center of the screen. Therefore, the distance of the occluder was 400 pixels. The velocity of the moving target varied from 133 (lower velocity), 200 (medium velocity), and 400 (higher velocity) pixels/s trial by trial, corresponding to three levels of actual TTC (TTCA): 3,000, 2,000, and 1,000 ms. Visual and auditory stimuli were presented at the same time, that is, visual and auditory stimuli were presented and disappeared simultaneously. The participants were explicitly told that the target maintained a constant velocity throughout the trial. Their task was to press a button when they estimated that the target contacted the destination of the occluder (Figure 1). The time between the stimulus disappearing and the button pressing was the TTC estimation (TTCE). If the participant did not respond within 5,000 ms, the trial was considered missing. The intertrial interval was 500 ms, and no feedback on the trial performance was given. Before the formal experiment, participants were asked to complete practice blocks for a few minutes with feedback.

Experiment 1 was a 2 (affective content: threat vs. non-threat) × 2 (sound: visual-only vs. audiovisual) × 3 (velocity: higher vs. medium vs. lower) design, resulting in 12 experimental conditions. Each condition was repeated 30 times, thus resulting in 360 trials. The formal experiment was divided into 6 × 60-trial blocks. For half of the participants, the first three blocks were visual-only conditions, and the second three blocks were audiovisual conditions. The different sound conditions were counterbalanced between the participants. For the other half of the participants, the first three blocks were audiovisual conditions, and the second three blocks were visual-only conditions. At the end of each block, participants were informed that they could rest for at least 1 min until they felt relief from their fatigue.

### 2.3. Results

Consistent with previous TTC studies (DeLucia and Liddell, 1998; Kiefer et al., 2006; Chotsrisuparat et al., 2017), the TTCE/TTCA ratio, i.e., the ratio of the TTCE and the TTCA, was calculated for each participant. The outlier trials in which the ratios exceeded 3 standard deviations in each velocity condition were excluded from further analysis (the overall effective rate of the data was higher than 98%). A TTCE/TTCA ratio higher than 100% indicated an overestimation of the TTC (i.e., pressing the button too late). In contrast, a TTCE/TTCA ratio lower than 100% indicated an underestimation of the TTC (i.e., pressing the button too early).

#### 2.3.1. TTCE/TTCA ratio

The TTCE/TTCA ratio in the 12 experimental conditions was entered into a 2 (affective content: threat vs. non-threat) × 2 (sound: visual-only vs. audiovisual) × 3 (velocity: higher vs. medium vs. lower) repeated measures analysis of variance (rmANOVA). Table 1 provides a summary of the mean TTCE/TTCA ratio under all conditions in Experiment 1. Here and in the subsequent analyses, an approach with the Greenhouse–Geisser correction for the degrees of freedom was used where applicable, and the value of *ε* is reported. *Post hoc* pairwise comparisons were conducted for significant interactions, using correction for multiple comparisons with Bonferroni correction.

**Table 1:** Mean TTCE/TTCA ratio (M ± SD) (%) in Experiment 1.

	Threat		Non-threat	
Velocity (TTCA)	Visual	Audiovisual	Visual	Audiovisual
Higher (1,000 ms)	146.44 (36.62)	145.14 (36.96)	147.23 (34.06)	152.76 (35.61)
Medium (2,000 ms)	113.69 (22.75)	109.32 (23.67)	114.39 (22.64)	112.98 (24.50)
Lower (3,000 ms)	91.05 (17.74)	87.41 (19.52)	89.93 (17.77)	88.42 (19.63)

The main effect of affective content was significant, *F*(1, 33) = 15.79, *p* < 0.001, *η_p_*^2^ = 0.32. The main effect of sound was not significant, *F* = 0.13, *p* = 0.72, *η_p_*^2^ = 0.004. The main effect of velocity was significant, *F*(1.08, 35.66) = 176.37, *p* < 0.001, *ε* = 0.54, *η_p_*^2^ = 0.84. The interaction between affective content and sound was significant, *F*(1, 33) = 21.66, *p* < 0.001, *η_p_*^2^ = 0.40. The interaction between affective content and velocity was significant, *F*(1.65, 54.60) = 7.69, *p* = 0.002, *ε* = 0.83, *η_p_*^2^ = 0.19. The interaction between sound and velocity was not significant, *F*(1.10, 36.27) = 2.19, *p* = 0.15, *ε* = 0.55, *η_p_*^2^ = 0.06. The three-way interaction among affective content, sound, and velocity was significant, *F*(1.51, 49.73) = 3.88, *p* = 0.038, *ε* = 0.75, *η_p_*^2^ = 0.11.

**Figure 2:**
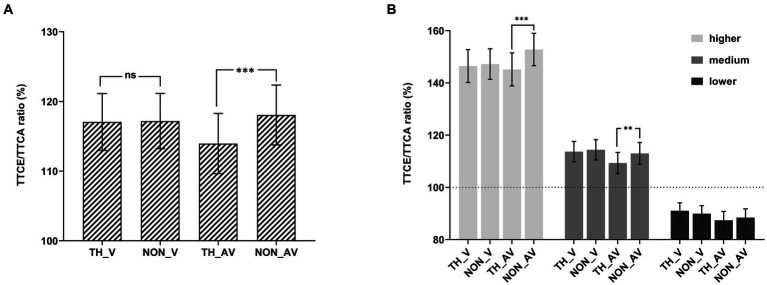
TTCE/TTCA ratio (%). The ratio shown as a function of the experimental conditions in Experiment 1. **(A)** Behavioral results of each condition by collapsing the three velocities. **(B)** Behavioral results of each condition. The error bars represent the standard errors of the mean. TH_V represents a visual threatening target without auditory information (visual-only), TH_AV represents a threatening target with auditory information (audiovisual), NON_V represents a visual non-threatening target without auditory information (visual-only), and NON_AV represents a non-threatening target with auditory information (audiovisual). ns *p* > 0.05, **p < 0.01, ***p < 0.001.

To explain the significant two-way interaction between affective content and sound, pairwise comparisons were conducted by collapsing the three velocities (see Figure 2A). Pairwise comparisons showed that the TTCE/TTCA ratio was significantly higher for audiovisual non-threatening targets (118.05%) than for audiovisual threatening targets (113.96%), *t*(33) = 6.01, *p_bonf_* < 0.001, Cohen’s *d* = 0.16, 95% CI = [2.24, 5.95]; while there was no significant difference between the visual non-threatening and visual threatening targets (visual-only condition), *t*(33) = 0.18, *p_bonf_* > 0.05.

To explore the three-way interaction, 2 (affective content: threat vs. non-threat) × 2 (sound: visual-only vs. audiovisual) rmANOVAs were performed separately for each velocity (see Figure 2B). For the higher velocity with the (1,000-ms TTCA) condition, the main effect of affective content was significant, *F*(1, 33) = 12.75, *p* = 0.001, *η_p_*^2^ = 0.28. The main effect of sound was not significant, *F*(1, 33) = 0.19, *p* = 0.67, *η_p_*^2^ = 0.006. The interaction between affective content and sound was significant, *F*(1, 33) = 16.46, *p* < 0.001, *η_p_*^2^ = 0.33. Pairwise comparisons showed that the TTCE/TTCA ratio was significantly higher for audiovisual non-threatening targets (152.76%) than for audiovisual threatening targets (145.14%), *t*(33) = 5.26, *p_bonf_* < 0.001, Cohen’s *d* = 0.21, 95% CI = [3.67, 11.56]; while there was no significant difference between the non-threatening and threatening targets in the visual-only condition, *t*(33) = 0.55, *p_bonf_* > 0.05.

For the medium velocity (2,000-ms TTCA) condition, the main effect of affective content was significant, *F*(1, 33) = 9.56, *p* = 0.004, η_p_^2^ = 0.23. The main effect of sound was not significant, *F*(1, 33) = 1.14, *p* = 0.29, *η_p_*^2^ = 0.03. The interaction between affective content and sound was significant, *F*(1, 33) = 4.81, *p* = 0.035, *η_p_*^2^ = 0.13. Pairwise comparisons showed that the TTCE/TTCA ratio was significantly higher for audiovisual non-threatening targets (112.98%) than for audiovisual threatening targets (109.32%), *t*(33) = 3.75, *p_bonf_* = 0.002, Cohen’s *d* = 0.15, 95% CI = [1.00, 6.31]; while there was no significant difference between the non-threatening and threatening targets in the visual-only condition, *t*(33) = 0.72, *p_bonf_* > 0.05.

For the lower velocity (3,000-ms TTCA) condition, the main effect of affective content was not significant, *F* = 0.02, *p* = 0.89, *η_p_*^2^ < 0.001. The main effect of sound was not significant, *F*(1, 33) = 1.55, *p* = 0.22, *η_p_*^2^ = 0.05. The interaction between affective content and sound was significant, *F*(1, 33) = 5.78, *p* = 0.022, *η_p_*^2^ = 0.15. Pairwise comparisons showed that the TTCE/TTCA ratio was not significant between threatening targets and non-threatening targets in the visual-only condition, *t*(33) = 1.92, *p_bonf_* > 0.05; there was no significant difference between the non-threatening and threatening targets in audiovisual condition, *t*(33) = 1.74, *p_bonf_* > 0.05.

#### 2.3.2. Central-tendency effect

To explain the significant main effect of the velocity condition, pairwise comparisons were conducted by collapsing the three velocities. Pairwise comparisons showed that the ratio was significantly higher for the higher velocity condition (147.89%) than for the medium velocity condition (112.594%), *t*(33) = 11.22, *p_bonf_* < 0.001, Cohen’s *d* = 1.27, 95% CI = [27.57, 43.03]; that the ratio was significantly higher for the higher velocity condition (147.89%) than for the lower velocity condition (89.20%), *t*(33) = 18.65, *p_bonf_* < 0.001, Cohen’s *d* = 2.23, 95% CI = [50.96, 66.42]; and that the ratio was significantly higher for the medium velocity condition (112.59%) than for the lower velocity condition (89.20%), *t*(33) = 7.44, *p_bonf_* < 0.001, Cohen’s *d* = 1.18, 95% CI = [15.66, 31.12].

To analyze whether there is a central-tendency effect in Experiment 1, we conducted a single-sample *t* test to compare the results with the ratio of the correct judgment (100%) under different velocity conditions. According to the single sample *t* test, we found that the ratio in the fast velocity condition (147.89%) was significantly higher than 100%, *t*(33) = 8.54, *p* < 0.001, Cohen’s *d* = 4.52, 95% CI = [36.48, 59.31]; the ratio in the medium velocity condition (112.59%) was significantly higher than 100%, *t*(33) = 3.36, *p* = 0.002, Cohen’s *d* = 5.15, 95% CI = [4.97, 20.22]; and the ratio in the lower velocity condition (89.20%) was significantly lower than 100%, *t*(33) = 3.58, *p* = 0.001, Cohen’s *d* = 5.07, 95% CI = [4.66, 16.94]. The results indicated that there was a significant overestimation effect under fast and medium velocity conditions, and a significant underestimation effect under slow velocity conditions; moreover, the overestimation effect under fast conditions was significantly higher than that under medium velocity conditions. This suggested that there was a central-tendency effect in Experiment 1.

### 2.4. Discussion

Experiment 1 aimed to study TTC estimation of threatening and non-threatening stimuli with auditory information. The results showed that participants underestimated the TTC of threatening stimuli in the audiovisual condition, but there was no significant difference between non-threatening targets with auditory information and without auditory information (DeLucia et al., 2014), as was the case with threatening targets. More specifically, for fast- and medium- velocity conditions (1,000- or 2,000- ms TTCA), the above results were replicated.

We found that TTC had a “threat advantage” effect in the audiovisual condition. Compared with other sensory systems, such as the visual and tactile systems, the auditory system, in particular, has several advantages (Posner et al., 1976; Klein, 1977; Juslin and Västfjäll, 2008). The auditory system can provide a more prominent continuous information flow and act as a warning system, which is more conducive to the perception of stimuli by participants. However, there were no significant differences between threatening and non-threatening targets in the visual-only condition, as the threat of facial expression was latent (DeLucia et al., 2014), and the emotional effect was relatively nonsignificant.

Furthermore, the TTC underestimation of audiovisual threatening targets was influenced by velocity. When the duration was the same, the lower the velocity, the more information the participants could extract, and the more accurately they could make judgments. Therefore, there were no differences between threatening stimuli and non-threatening stimuli. The higher the velocity, the more challenging it was for participants to make judgments, and the more likely it was to distort time perception, resulting in the TTC underestimation of threatening stimuli. According to scalar timing theory, during the clock stage, the accumulator collects the pulses emitted by the pacemaker (Gibbon, 1977; Gibbon et al., 1984). The number of pulses collected by the accumulator represents the time duration of a particular interval. When people encounter a threat stimulus, the affective content of the stimulus increases the frequency of the pulses emitted by the pacemaker, so the duration perception is prolonged; thus, individuals underestimate TTC.

When considering the results of different velocity conditions, we found that there was a potential trend of the “central-tendency effect.” Specifically, the results showed an underestimation effect of the lower velocity (3,000-ms TTCA) condition and an overestimation effect of the higher velocity (1,000-ms TTCA) condition. TTCE was as accurate (or nearly so) as that of the medium velocity (2,000-ms TTCA) condition. Researchers regarded the phenomenon as noise that interfered with an accurate measure of the psychophysical scale or as distortion that revealed psychological processes underlying mental representations (Lejeune and Wearden, 2009). To eliminate or reduce this effect, previous studies used a grouped design, i.e., standard durations are presented in a grouped design in which the order of presentation of the standard duration blocks is random (Ryan, 2012, 2016). To preclude the confounds of such a central tendency effect, we conducted Experiment 2, in which the velocity condition was presented in groups.

## 3. Experiment 2: auditory affective content affects different velocities of grouped TTC estimation

### 3.1. Method

#### 3.1.1 Participants

Thirty-four participants (mean age 22.01 ± 2.8 years, range 18–26, 23 females) participated in Experiment 2. All participants were right-handed and had normal or corrected-to-normal vision and color vision and none of them had a history of neurological or psychiatric disorders, as self-reported. Informed consent was obtained from each participant before the experiment was conducted according to the Declaration of Helsinki before their inclusion. They had not participated in similar experiments before. The study was approved by the Academic Committee of the Department of Psychology, Soochow University, China.

To evaluate the statistical testing power in the present study, sensitivity analysis was conducted on the two-side paired *t* test through the software G*Power 3.1 (Faul et al., 2007). The input parameters were as follows: *α* err prob = 0.05, power (1-*β* err prob) = 0.80, and total sample size = 34. The output parameters of Cohen’s *d* = 0.50 were calculated, which reached a medium effect size.

#### 3.1.2. Apparatus, stimuli, and experimental setup

In Experiment 2, the apparatuses and stimuli and the experimental setup matched those in Experiment 1.

### 3.2. Experimental procedures and design

In Experiment 2, the experimental steps were the same as those described in Experiment 1. The only exception was that the velocity condition was presented in a grouped manner. Each block consisted of five groups (60 trials in total), and each group consisted of 12 trials with the same velocity. For each group, we informed the participant that the velocity of the next group would change. Because there were three-velocity conditions, we used the “Latin square design” to counterbalance the condition. Experiment 2 was also a 2 (affective content: threat vs. non-threat) × 2 (sound: visual-only vs. audiovisual) × 3 (velocity: higher vs. medium vs. lower) design, resulting in 12 experimental conditions. Each condition was repeated 30 times, thus resulting in 360 trials. The formal experiment was divided into 6 × 60-trial blocks.

### 3.3. Results

The outlier trials in which the ratios exceeded three standard deviations in each velocity condition were excluded from further analysis (the overall effective rate of the data was higher than 98%).

#### 3.3.1. TTCE/TTCA ratio

The TTCE/TTCA ratio in the 12 experimental conditions was entered into a 2 (affective content: threat vs. non-threat) × 2 (sound: visual-only vs. audiovisual) × 3 (velocity: higher vs. medium vs. lower) rmANOVA. Table 2 provides a summary of the mean TTCE/TTCA ratio under all conditions in Experiment 2.

**Table 2:** Mean TTCE/TTCA ratio (M ± SD) (%) of the means in Experiment 2.

	Threat		Non-threat	
Velocity (TTC)	Visual	Audiovisual	Visual	Audiovisual
Higher (1,000 ms)	121.37 (30.02)	129.06 (35.97)	122.16 (28.96)	133.44 (35.04)
Medium (2,000 ms)	112.53 (21.71)	113.90 (16.77)	112.88 (20.39)	118.01 (17.07)
Lower (3,000 ms)	94.05 (17.55)	95.11 (14.33)	96.63 (16.70)	96.01 (14.21)

The 2 (affective content: threat vs. non-threat) × 2 (sound: visual-only vs. audiovisual) × 3 (velocity: higher vs. medium vs. lower) rmANOVA showed that the main effect of affective content was significant, *F*(1, 33) = 15.14, *p* < 0.001, *η_p_*^2^ = 0.31. The main effect of sound was significant, *F*(1, 33) = 4.69, *p* = 0.038, *η_p_*^2^ = 0.12. The main effect of velocity was significant, *F* (1.19, 39.13) = 33.19, *p* < 0.001, *ε* = 0.59, *η_p_*^2^ = 0.50. The interaction between affective content and sound was significant, *F*(1, 33) = 8.77, *p* = 0.006, *η_p_*^2^ = 0.21, as were the interaction between sound and velocity, *F*(1.53, 50.32) = 5.27, *p* = 0.014, *ε* = 0.76, *η_p_*^2^ = 0.14. The interaction between affective content and velocity was marginally significant, *F*(1.60, 52.93) = 3.11, *p* = 0.063, *ε* = 0.80, *η_p_*^2^ = 0.09. The three-way interaction was not significant, *F*(1.71, 56.33) = 1.21, *p* = 0.30, *ε* = 0.85, *η_p_*^2^ = 0.04.

**Figure 3:**
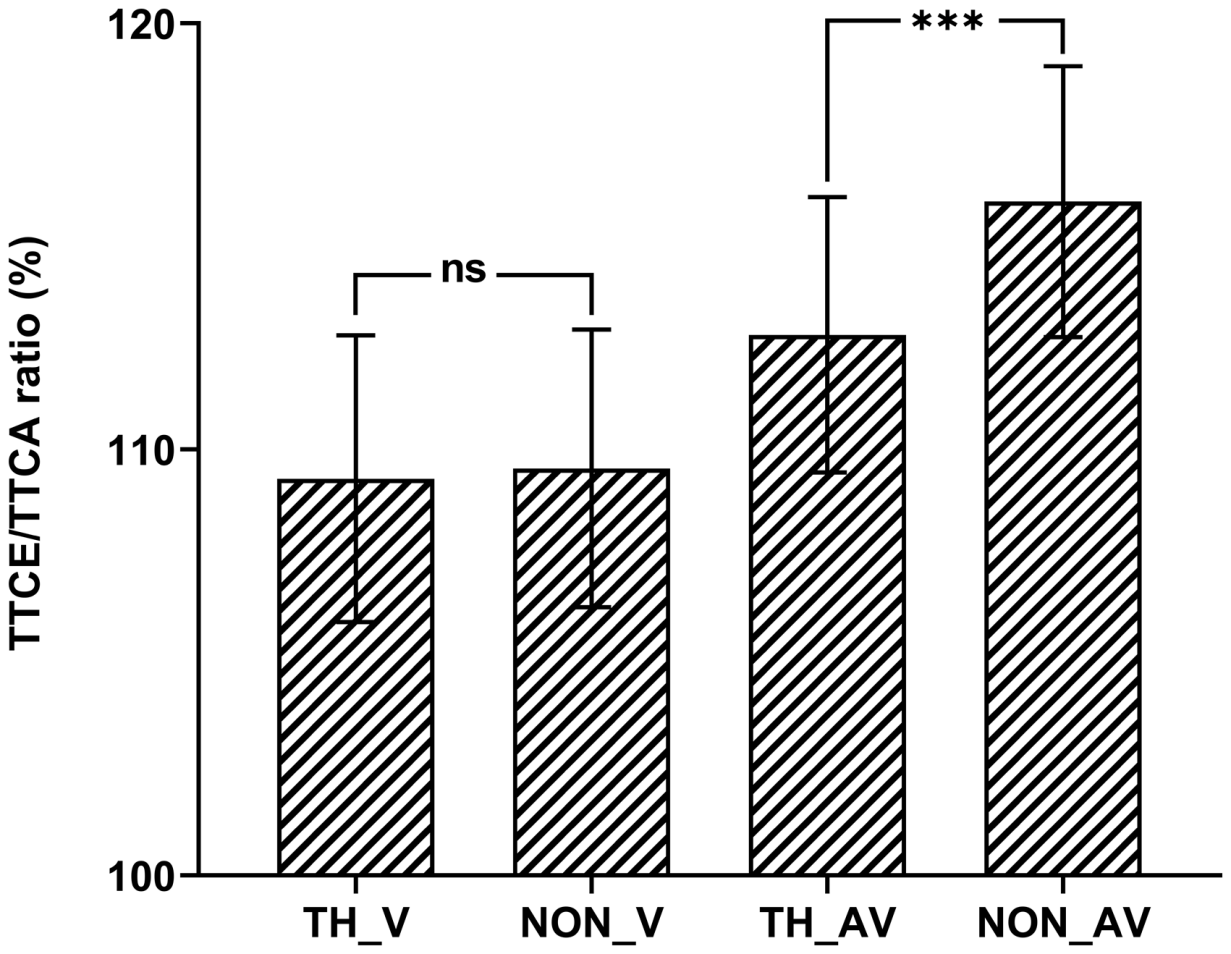
TTCE/TTCA ratio (%). Shown as a function of the experimental conditions in Experiment 2. Behavioral results of each condition by collapsing the three velocity. ns *p* > 0.05, ***p < 0.001.

To explain the significant two-way interaction between affective content and sound, pairwise comparisons were conducted by collapsing the three velocities (see Figure 3). The result showed that the TTCE/TTCA ratio was significantly higher for audiovisual non-threatening targets (115.82%) than for audiovisual threatening targets (112.69%), *t*(33) = 4.80, *p_bonf_* < 0.001, Cohen’s *d* = 0.17, 95% CI = [1.35, 4.90]; while there was no significant difference between the non-threatening and threatening targets in the visual-only condition, *t*(33) = 0.38, *p_bonf_* > 0.05.

#### 3.3.2. Central-tendency effect

To explain the significant main effect of velocity condition, pairwise comparisons were conducted by collapsing the three velocities. Pairwise comparisons showed that the ratio was significantly higher for the higher velocity condition (126.51%) than for the medium velocity condition (114.33%), *t*(33) = 3.09, *p_bonf_* = 0.009, Cohen’s *d* = 0.48, 95% CI = [2.50, 21.86]; that the ratio was significantly higher for the higher velocity condition (126.51%) than for the lower velocity condition (94.70%), *t*(33) = 8.07, *p_bonf_* < 0.001, Cohen’s *d* = 1.31, 95% CI = [22.13, 41.48]; that the ratio was significantly higher for the medium velocity condition (114.33%) than for the lower velocity condition (94.70%), *t*(33) = 4.98, *p_bonf_* < 0.001, Cohen’s *d* = 1.23, 95% CI = [9.95, 4.98].

To analyze whether there is a central-tendency effect in the Experiment 2, we conducted single-sample *t-*test to compare the results with the ratio of the correct judgment (100%) under different velocity conditions. According to the single sample *t*-test, we found that the ratio of fast velocity condition (126.51%) was significantly higher than 100%, *t*(33) = 4.97, *p* < 0.001, Cohen’s *d* = 4.07, 95% CI = [15.65, 37.36]; the ratio of medium velocity condition (114.33%) was significantly higher than 100%, *t*(33) = 4.83, *p* < 0.001, Cohen’s *d* = 6.62, 95% CI = [8.30, 20.36]; the ratio of lower velocity condition (94.7%) was significantly lower than 100%, *t*(33) = 2.15, *p* = 0.039, Cohen’s *d* = 6.58, 95% CI = [0.27, 10.32]. This suggests that there also had a central-tendency effect in Experiment 2.

To explore whether the central-tendency effect was reduced in Experiment 2 by changing the presentation manner of Experiment 2, we conducted a mixed design of 2 (velocity: velocity: higher vs. medium vs. lower) × 2 (experiment: Experiment 1 vs. Experiment 2) rmANOVA. The main effect of velocity was significant, *F*(1.15, 75.58) = 161.25, *p* < 0.001, *ε* = 0.57, *η_p_*^2^ = 0.71. The main effect of experiment was not significant, *F*(1, 66) = 0.92, *p* = 0.34, *η_p_*^2^ = 0.01. The interaction between velocity and experiment was significant, *F*(2, 132) = 16.68, *p* < 0.001, *η_p_*^2^ = 0.20. We further used the pairwise comparisons to compare the ratio under different velocity conditions in Experiment 1 and Experiment 2. The results showed that the ratio was significantly higher for Experiment 1 (147.89%) than for Experiment 2 (112.59%) in the higher velocity condition, *t*(33) = 3.37, *p_bonf_* = 0.02, Cohen’s *d* = 0.67, 95% CI = [1.93, 40.81], indicating that the overestimation effect induced by the central-tendency was significantly reduced; the ratio was not significant between the Experiment 1 (112.5%) and Experiment 2 (114.33%) in the medium velocity condition, *t*(33) = 2.29, *p_bonf_* > 0.05; in the lower velocity condition, the underestimation effect induced by the central-tendency was smaller in Experiment 2 (94.70%) than in Experiment 1 (89.20%), although not reached significance, *t*(33) = 0.87, *p_bonf_* > 0.05. These results indicated that, by controlling the presentation manner of Experiment 2, the central-tendency effect was reduced.

### 3.4. Discussion

In Experiment 1, we found a trend of the central-tendency effect in TTC studies by control velocity, which is consistent with the findings of previous studies (Li et al., 2015). To decrease the specific trend of the central-tendency effect, we controlled the presentation of stimuli in a grouped manner and conducted the control experiment of Experiment 2 instead of the trial-by-trial manner of Experiment 1 (Ryan, 2012, 2016). With the control allowed by the grouped approach, the central-tendency effect was reduced.

From the perspective of time perception, the “central-tendency effect” is one of the characteristics of time perception. The Internal Reference Model (IRM) assumes an internal standard reference interval in the brain, which is weighted and updated each time the brain makes a judgment based on the internal parameter data obtained from the previous experiment and the current experiment interval (Bausenhart et al., 2014; Polti et al., 2018). Therefore, when presenting several durations, people would overestimate the shorter durations and underestimate the longer durations. More specifically, the “central-tendency effect” in Experiment 1 is the “velocity-induced time dilation effect.” The velocity also affects time perception, where an increase in velocity can lead to overestimations of durations, and vice versa (Matthews, 2011; Karşılar et al., 2018). Since the target motion can be defined in terms of change per unit time (Poynter, 1989), when the target disappears, more changes occur per unit time when the velocity is higher, thus leading to an overestimation of duration (Kanai et al., 2006; Kaneko and Murakami, 2009).

In summary, when precluding the potential confounds of the central tendency, the results of Experiment 2 replicated the main findings of Experiment 1: threatening audiovisual targets facilitated TTC estimation. As stated in the Introduction, the presentation time of the target is positively correlated with the TTC estimation, which means that a longer presentation time improves performance (Peterken et al., 1991; Battaglini and Ghiani, 2021). However, this conclusion does not take the effect of velocity into account, so a comparison of the interaction between the velocity of stimulus and presentation time is needed. To compare the influence of stimulus velocity and presentation time on TTC estimation, we conducted Experiment 3. In Experiment 3, we explored the influence of different presentation times on TTC estimation of threat stimuli.

## 4. Experiment 3: auditory affective content affects TTC estimation at different presentation time

In Experiment 1 and Experiment 2, we manipulated the velocity condition by varying the velocity of the moving target. To reveal the differential effects of velocity and presentation time on TTC estimation, in Experiment 3, we varied the presentation time of the moving target resulting in different TTCA which matched those in Experiment 1 and Experiment 2.

### 4.1. Method

#### 4.1.1. Participants

Thirty-four participants (mean age 21.76 ± 2.21 years, range 19 to 25, 26 females) participated in Experiment 3. All participants were right-handed and had normal or corrected-to-normal vision and color vision and none of them had a history of neurological or psychiatric disorders. Informed consent was obtained from each participant according to the Declaration of Helsinki. They had not participated in similar experiments before. The study was approved by the Academic Committee of the Department of Psychology, Soochow University, China.

To evaluate the statistical testing power in the present study, sensitivity analysis was conducted on the two-side paired *t-*test through the software G*Power 3.1 (Faul et al., 2007). The input parameters were as follows: *α* err prob = 0.05, power (1-*β* err prob) = 0.80, and total sample size = 34. The output parameters of Cohen’s *d* = 0.50 were calculated, which reached a medium effect size.

#### 4.1.2. Apparatus, stimuli, and experimental setup

The apparatuses and facial expressions of Experiment 3 were consistent with those in Experiment 1 and Experiment 2. The parameters and processing methods of auditory stimuli were consistent with those of experiment 1. All the auditory stimuli were lasting for 2,500 ms.

### 4.2. Experimental procedures and design

Each trial began with a 500-ms fixation period followed by the presentation of the target. The target moved leftward for 1,000-, 600-, and 200-pixels beginning from 550 pixels to the right of the screen center until it reached the right edge of an occluder and disappeared. The participants were told that although the target disappeared, it would continue moving behind the occluder until it collided with the left edge of the occluder, which was a thin vertical red line 850 pixels to the left of the screen center. Therefore, the distance between the right and left edges of the occluder varied (i.e., 1,200-, 800- and 400- pixels), while the target’s velocity was constant at 400 pixel/s. The presentation time of the moving target was 500, 1,500, and 2,500 ms, corresponding to three levels of TTCA of 3,000, 2000, and 1,000 ms. We played the voice stimuli for a corresponding amount of time duration according to the stimulus presentation time. Since the velocity of the visual target was consistent in Experiment 3, the range of voice stimuli stimulation presentation did not change. The other parameters of auditory stimuli were presented in the same way as in Experiment 1. The participants were told that the target maintained a constant velocity throughout the trial. Participants were to press a button when they thought that the target collided with the left edge of the occluder (Figure 1A). If the participant did not respond within 5,000 ms, the trial was considered missing. The intertrial interval was 500 ms, and no feedback on the trial performance was given. Before the formal experiment, participants were asked to complete practice blocks for a few minutes with feedback.

Experiment 3 was a 2 (affective content: threat vs. non-threat) × 2 (sound: visual-only vs. audiovisual) × 3 (presentation time: longer vs. medium vs. shorter) design, resulting in 12 experimental conditions. Each condition was repeated 30 times, thus resulting in 360 trials. The formal experiment was divided into 6 × 60-trial blocks. Different participants and different sound conditions were counterbalanced and consistent with those in Experiment 1.

### 4.3. Results and discussion

The outlier trials in which the ratios exceeded 3 standard deviations in each velocity condition were excluded from further analysis (the overall effective rate of the data was higher than 98%).

#### 4.3.1. TTCE/TTCA ratio

The TTCE/TTCA ratio in the 12 experimental conditions was entered into a 2 (affective content: threat vs. non-threat) × 2 (sound: visual-only vs. audiovisual) × 3 (presentation time: longer vs. medium vs. shorter) rmANOVA. Table 3 provides a summary of the mean TTCE/TTCA ratio under all conditions in Experiment 3.

**Table 3:** Mean TTCE/TTCA ratio (M ± SD) (%) of the means in Experiment 3.

Presentation time (TTC)	Threat		Non-threat	
	Visual	Audiovisual	Visual	Audiovisual
Longer (1,000) ms	126.43 (26.41)	122.36 (19.24)	126.20 (25.08)	126.43 (19.14)
Medium (2,000) ms	123.33 (19.94)	122.02 (16.17)	123.01 (19.17)	122.29 (15.25)
Shorter (3,000) ms	115.88 (16.12)	116.92 (15.13)	115.68 (16.39)	116.59 (14.60)

**Figure 4:**
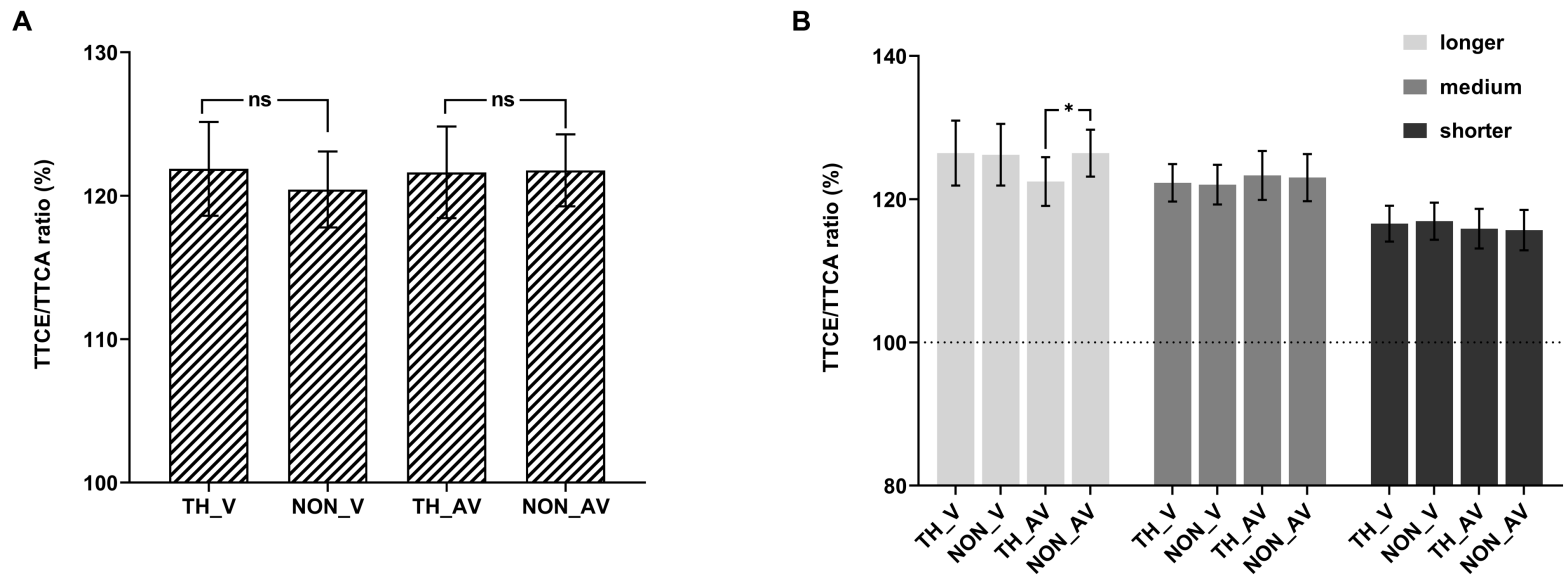
TTCE/TTCA ratio (%). The ratio shown as a function of the experimental conditions in Experiment 3. **(A)** Behavioral results of each condition by collapsing the three velocities. **(B)** Behavioral results of each condition. The error bars represent the standard errors of the mean. *p < 0.05, ns *p* > 0.05.

The 2 (affective content: threat vs. non-threat) × 2 (sound: visual-only vs. audiovisual) × 3 (presentation time: longer vs. medium vs. shorter) rmANOVA showed that the main effect of affective content was not significant, *F*(1, 33) = 1.01, *p* = 0.32, *η_p_*^2^ = 0.03. The main effect of sound was not significant, *F*(1, 33) = 0.08, *p* = 0.78, *η_p_*^2^ = 0.002. The main effect of presentation time was significant, *F*(1.22, 40.23) = 8.98, *p* = 0.003, *ε* = 0.61, *η_p_*^2^ = 0.21. The interaction between affective content and sound was significant, *F*(1, 33) = 4.22, *p* = 0.048, *η_p_*^2^ = 0.11, pairwise comparisons showed that there was no difference between threat and non-threat (see Figure 4A). The interaction between affective content and presentation time was significant, *F*(1, 33) = 3.13, *p* = 0.05, *η_p_*^2^ = 0.09. The interaction between sound and presentation time was not significant, *F*(1.40, 46.13) = 1.77, *p* = 0.19, *ε* = 0.70, *η_p_*^2^ = 0.05. The three-way interaction was significant, *F*(1.69, 55.60) = 3.95, *p* = 0.031, *ε* = 0.84, *η_p_*^2^ = 0.11.

In order to explain the significant three-way interaction, two by two ANOVAs were conducted separately for each presentation time level (see Figure 4B). For the longer presentation time (1,000-ms TTCA) condition, the main effect of affective content was marginally significant, *F*(1, 33) = 3.07, *p* = 0.089, *η_p_*^2^ = 0.09. The main effect of sound was not significant, *F* = 0.43, *p* = 0.52, *η_p_*^2^ = 0.01. The interaction between affective content and sound was significant, *F*(1, 33) = 7.16, *p* = 0.012, *η_p_*^2^ = 0.18. Pairwise comparisons showed that the TTCE/TTCA ratio was significantly higher for audiovisual non-threatening targets (126.43%) than for audiovisual threatening targets (122.36%), *t*(33) = 2.30, *p_bonf_* = 0.024, Cohen’s *d* = 0.21, 95% CI = [0.36, 7.78]; while there was no significant difference between the non-threatening and threatening targets in the visual-only condition, *t*(33) = 0.17, *p_bonf_* > 0.05.

For the medium presentation time (2,000-ms TTCA) condition, all the main effect and interaction among the 2 (affective content) by 2 (sound) ANOVA were not significant. The main effect of affective content was not significant, *F*(1, 33) = 0.001, *p* = 0.98, *η_p_*^2^ < 0.001. The main effect of sound was not significant, *F* = 0.17, *p* = 0.68, *η_p_*^2^ = 0.01. The interaction between affective content and sound was not significant, *F*(1, 33) = 0.23, *p* = 0.64, *η_p_*^2^ = 0.01.

For the shorter presentation time (3,000-ms TTCA) condition, all the main effect and interaction among the 2 (affective content) by 2 (sound) ANOVA were not significant. The main effect of affective content was not significant, *F*(1, 33) = 0.42, *p* = 0.52, *η_p_*^2^ = 0.01. The main effect of sound was not significant, *F* = 0.25, *p* = 0.62, *η_p_*^2^ = 0.01. The interaction between affective content and sound was not significant, *F*(1, 33) = 0.03, *p* = 0.87, *η_p_*^2^ < 0.001.

#### 4.3.2. Central-tendency effect

To explain the significant main effect of the presentation time condition, pairwise comparisons were conducted by collapsing the three presentation times. Pairwise comparisons showed that the ratio was no significant difference between the longer presentation time condition and the medium presentation time condition, *t*(33) = 1.22, *p_bonf_* > 0.05; that the ratio was significantly higher for the longer presentation time condition (125.35%) than for the shorter presentation time condition (116.27%), *t*(33) = 4.13, *p_bonf_* < 0.001, Cohen’s *d* = 0.51, 95% CI = [3.68, 14.50]; that the ratio was significantly higher for the medium presentation time condition (122.66%) than for the shorter presentation time condition (112.66%), *t*(33) = 2.90, *p_bonf_* = 0.015, Cohen’s *d* = 0.42, 95% CI = [0.98, 11.81]. We found all three presentation time conditions were overestimated, and there is no significant difference between the longer and the medium presentation time condition, thus indicating there was no central-tendency effect and the effect in Experiment 1 was a velocity-induced time dilation effect.

### 4.4. Discussion

Based on the findings of Experiment 1, we want to explore the influence of presentation time on TTC estimation. Overall, differences between the threatening and non-threatening targets were found only in the 2,500-ms presentation time (1,000-ms TTCA) condition with the addition of auditory information, and the threatening auditory information facilitated the participants’ judgments. With the addition of auditory information, the difference between threatening stimuli and non-threatening stimuli could still be found when the presentation time was relatively long under fast conditions, possibly because a longer presentation time can extract more target motion information when the velocity is fast. Therefore, it seemed that velocity was a more critical factor for the threatening facilitation effect.

The threatening facilitation effect was found in both the 1,000-ms TTCA conditions in Experiment 1 (400-pixel/s velocity and 1,500-ms presentation time) and Experiment 3 (400-pixel/s velocity and 2,500-ms presentation time) with the addition of auditory information (same velocity but different presentation time). However, such an effect was found only in the 2,000-ms TTCA condition in Experiment 1 (200-pixel/s velocity and 1,500-ms presentation time) but not in the 2,000-ms TTCA condition in Experiment 3 (400-pixel/s velocity and 1,500-ms presentation time) (same presentation time but different velocity). These results indicate that differences between threatening stimuli and non-threatening stimuli can be found when the presentation time is relatively long. As mentioned above, the rapid response is due to the short presentation time and the smaller amount of information that the participants obtained, not simply because the target stimuli moved higher (Peterken et al., 1991; Mason and Carnahan, 1999). Chang and Jazayeri (2018) found that when explicit timing cues were available, participants combined time information with velocity to obtain more accurate estimation, suggesting that humans improve their performance by integrating information from multiple patterns (Ernst and Banks, 2002; Hillis et al., 2002). The results also showed that the integration of velocity and time information is different from the indirect effect of increasing presentation time to improve velocity estimation. People actively integrate multiple pieces of information on velocity and time to intercept the target to obtain a more accurate TTC estimation (Chang and Jazayeri, 2018; Chang, 2021).

## 5. General discussion

When participants estimate TTC, there are differences in the TTC estimation between threatening stimuli and non-threatening stimuli, as TTC has a “threat advantage” effect (Brendel et al., 2012; Vagnoni et al., 2012; Delucia et al., 2014). Here, we aimed to study the visual or audiovisual TTC estimation of threatening stimuli in the transversal TTC task paradigm. The results showed that (1) the addition of auditory affective content facilitated TTC estimation; (2) by presenting the velocity condition in a grouped manner, we precluded the confounds of the central-tendency effect; and (3) by comparing the findings of Experiment 1 with those of Experiment 3, we found that velocity played a more crucial role in TTC estimation than presentation time.

### 5.1. Auditory affective content facilitates performance in TTC estimation

According to our results, we found that threats facilitated TTC estimation in the audiovisual conditions in Experiment 1 and Experiment 2, and the TTC estimation of non-threatening targets was higher than that of threatening targets. This effect was replicated in the higher and medium velocity conditions (1,000- and 2,000-ms TTCA). However, at lower velocities, the threat advantage disappeared.

Consistent with the visual domain in which threatening targets influence TTC estimation, it is reasonable to expect that a similar mechanism exists in the auditory domain. To clarify, the use of multiple modalities does not imply that people have equal aptitude for using that information across all modalities. The auditory system has many advantages over other sensory systems (i.e., the visual system and tactile system), suggesting that it serves as an alert or warning system (Posner et al., 1976; Klein, 1977; Juslin and Västfjäll, 2008) and is thus sensitive to threats. Gordon and Rosenblum (2005) suggest that, for speech, it is often the case that the acoustic medium facilitates perception better than the visual medium. It may be threatening auditory information that induces a negative effect that would influence audiovisual spatial judgments and accelerate TTC estimation (Neuhoff et al., 2014). Therefore, we found that participants underestimated TTC threatening stimuli with auditory information.

The effect of auditory information facilitating TTC estimation under higher and medium velocity conditions can be explained from the “time perception” and “velocity perception” perspectives. On the one hand, from the perspective of time perception, our results can be explained by scalar timing theory. Scalar timing theory consists of three sequential stages: the clock stage, the memory stage, and the decision-making stage (Gibbon, 1977; Gibbon et al., 1984; Karşılar et al., 2018; Tian et al., 2018b). During the clock stage, the accumulator collects the pulses emitted by the pacemaker, and the number of pulses collected by the accumulator represents the time duration of a particular interval. The switch controls pulse transmission from the pacemaker to the accumulator. When the switch is off, the pulse enters the accumulator; when the switch is on, the pulse is blocked externally. In the memory stage, the time duration is transferred from working memory to long-term memory. Finally, in the decision-making stage, the duration being timed is compared in a person’s mind with a representation of the duration stored in long-term memory to determine if the current interval has the same duration. When people encounter a threat stimulus, the affective content of the stimulus increases the pulse rate emitted by the pacemaker, resulting in prolonged duration perception (Droit-Volet et al., 2013; Schwarz et al., 2013). In the fast and medium velocity conditions, compared with the non-threatening stimuli, participants may not have an illusion of altered time. The participants judged the presentation time of threatening stimuli to be longer than that of non-threatening stimuli, so they would have a higher response after the stimulus disappeared, which was shown as TTC underestimation. Under slow conditions, the prominence of the threatening stimuli is reduced, so the pulses emitted do not differ from the non-threatening stimuli and do not show TTC underestimation.

On the other hand, from the perspective of velocity perception, individuals underestimated TTC threatening visual and auditory stimuli probably because participants overestimated the moving velocity of stimuli, and the higher the velocity, the greater the proportion of underestimation. Riskind et al. (1995) investigated individuals’ motion perception of spider pictures, and the results showed that groups suffering from arachnophobia felt venomous spiders moving toward them faster than groups without arachnophobia (Riskind et al., 1995). Moreover, according to structuralist theory, distance is equal to the product of time and velocity, more changes occur per unit time when the velocity is higher (Poynter, 1989). In the experiment, TTC estimation was made faster when participants’ subjective perception of threat and visual and auditory stimuli moved faster. However, under the slow condition, the lower-moving threat stimulus did not make the participants perceive the threat, so they did not overestimate their moving velocity.

It is worth noting that we did not find that threats facilitated TTC estimation in the visual conditions in Experiment 1 and Experiment 2. We speculate that there are some possibilities. First, according to Bar et al. (2006), emotionally neutral faces can be judged to be threatening. Previous studies used angry faces, neutral faces, and happy faces, and they found that participants underestimated TTC for threatening targets (Brendel et al., 2012; Silvestri et al., 2022), but we did not use neutral facial expressions. This may indicate that there is a comparative effect on emotional TTC estimation, i.e., shorter estimation for angry faces, longer estimation for happy faces, and intermediate estimation for neutral faces. We did not use neutral facial expressions in our study, which may have resulted in no difference between the angry faces and happy faces (Delucia et al., 2014). Second, we used angry expressions to elicit threat-related responses (Reed et al., 2014; Reilly et al., 2022), but the potential threat of facial expressions is relatively weak (DeLucia et al., 2014). It is merely implicit in the emotional term, so the effect of threat facilitating TTC estimation is unstable, and the effect of emotional stimulation is not obvious. A threatening facial expression implies a variety of possible outcomes including different degrees of threats and even nonthreats, and the assessment of facial expression threats from social groups is more flexible and controlled more by cognitive factors.

### 5.2. Integration of velocity and time in TTC estimation

Our results seemed to demonstrate that velocity was a critical factor for the threatening facilitation effect. The results also showed that the integration of velocity and time information is different from the indirect effect of increasing presentation time that leads to improved velocity estimation and that people actively integrate velocity and time to intercept the target (Chang and Jazayeri, 2018; Chang, 2021).

Our results indicated that differences between threatening stimuli and non-threatening stimuli can be found when the presentation time is relatively long. Mason and Carnahan (1999) used a presentation time of 1,000 ms and found that the TTC estimation of the target did not vary with the target velocity, which is possible because the presentation time was relatively short. Subsequently, Tresilian and Lonergan (2002) found that a relatively long presentation time (1,000–2,000 ms) would affect the accuracy of TTC estimation, indicating that after controlling the velocity, the presentation time would have a corresponding impact on TTC estimation. In general, people can extract more target motion information when the velocity is fast with a longer presentation time. People’s responses are limited by fast velocity, which makes the estimation task more challenging, and the longer the duration time is, the more accurate the response. Chang and Jazayeri (2018) used the Bayesian model to effectively integrate the prior knowledge of TTC with velocity and time cues to predict behavioral responses. The human brain is optimized for integrating velocity and time information for TTC estimation, and this optimization indicates that this integration is prioritized over the indirect effect of changing velocity. However, it is worth noting that the authors also pointed out that the velocity estimation becomes saturated as the presentation time increases, so it is not sufficient to simply increase the velocity or presentation time.

### 5.3. Central-tendency effect of TTC estimation

We found a central-tendency effect in Experiment 1 and Experiment 2 rather than in Experiment 3. This suggested that this central-tendency effect is a velocity-induced time dilation effect. Consistent with previous studies, as the stimulus velocity slows down, the proportion of overestimated TTC increases (Li et al., 2015). It was found that a significantly overestimated effect was observed at high velocity, and a significantly underestimated effect was observed at low velocity (Kaneko and Murakami, 2009; Makin et al., 2012; Block and Gruber, 2014). This result can be explained from two aspects “time perception” (Polti et al., 2018) and “velocity perception” (Karşılar et al., 2018).

From the perspective of time perception, the characteristics of time perception consist of the “central-tendency effect,” “range effect” and “scalar variability.” The central tendency effect is a signature of Bayesian computations in the estimation of a duration time; the longer the duration time is, the more obvious the central tendency. Researchers have suggested that people rely on IRM when making judgments about which hypothesis people use to build up an internal standard reference (Bausenhart et al., 2014). People make a judgment based on the internal parameter data of the previous experiment and the current experiment interval (Bausenhart et al., 2014; Polti et al., 2018). Therefore, in the time perception task, people weighed various lengths of time duration so that they differed from the judgment standard interval. When the target disappeared, occlusion distance = time × velocity, fast velocity corresponds to a short time, and lower velocity corresponds to a long time. In our experiment, different TTC estimations formed a time range, so people’s estimates gradually approached the central mean. The results showed an overestimation effect under higher-velocity conditions corresponding to a short TTCA, while a significant underestimation effect under lower-velocity conditions corresponded to a long TTCA.

From the perspective of velocity perception, velocity also affects time perception and reflects the “velocity-induced time dilation effect” (Matthews, 2011; Karşılar et al., 2018); a faster moving target introduces a longer subjectively experienced temporal duration than a slower target (Li et al., 2015). On the one hand, the mechanism can be explained by scalar timing theory. In the clock stage, the pacemaker emits pulses, which are then collected by an accumulator, and the number of pulses collected by the accumulator represents the duration. At higher velocities, more pulses accumulate per unit time in the accumulator and thus accelerate the pacemaker, resulting in a longer perceptual duration, as shown by TTC overestimation. At lower velocities, fewer pulses accumulate per unit time in the accumulator, slowing down the pacemaker and resulting in a shorter perception duration, as shown by TTC underestimation. On the other hand, this outcome can also be explained by the neural energy model of timing. Because neural response reflects the metabolic cost of neural information processing (“neural energy”), Pariyadath and Eagleman (2008) proposed that the strength of neural response may be the determinant of the perceived duration. According to Kaufmann et al. (2000), when participants observed faster-walking velocity, stronger neural activation was observed, which meant more neural processing. According to the neural energy model, this leads to longer time estimates, represented by a higher rate of overestimation with higher velocity and vice versa.

## 6. Conclusion

In summary, by adding the affective content of auditory voices to the visual TTC estimation, we found that participants underestimated the TTC of audiovisual threatening targets. By controlling the presentation manner of velocity, the central-tendency effect was reduced. Furthermore, by controlling velocity and presentation time, we found differences between the same velocity and different presentation times. However, there were no differences between the same presentation time and different velocities, which suggests that the velocity was a more critical factor for the audiovisual threatening facilitation effect. According to our results, we should pay more attention to the velocity of moving targets and auditory information, which provide more important information about how these factors may affect daily life.

## Data availability statement

The raw data supporting the conclusions of this article will be made available by the authors, without undue reservation.

## Ethics statement

The study was approved by the Academic Committee of the Department of Psychology, Soochow University, China. The patients/participants provided their written informed consent to participate in this study. Written informed consent was obtained from the individual(s) for the publication of any potentially identifiable images or data included in this article.

## Author contributions

All authors listed have made a substantial, direct, and intellectual contribution to the work and approved it for publication.

## Funding

This research was supported by the Suzhou Science and Technology Development Plan (People’s Livelihood Science and Technology: SKY2022113), the Humanities and Social Sciences Research Project of Soochow University (22XM0017), Interdiscipline Research Team of Humanities and Social Sciences of Soochow University (2022), the National Natural Science Foundation of China (31871092), the National Social Science Foundation of China (22CYY018), the Japan Society for the Promotion of Science KAKENHI (20K04381), and the JST FOREST Program (JPM-JFR2041).

## Conflict of interest

The authors declare that the research was conducted in the absence of any commercial or financial relationships that could be construed as a potential conflict of interest.

## Publisher’s note

All claims expressed in this article are solely those of the authors and do not necessarily represent those of their affiliated organizations, or those of the publisher, the editors and the reviewers. Any product that may be evaluated in this article, or claim that may be made by its manufacturer, is not guaranteed or endorsed by the publisher.
