# Supplementary material for: Auditory affective content facilitates time-to-contact estimation of visual affective targets
Source: Front Psychol. 2023 May 3;14:1105824. doi: 10.3389/fpsyg.2023.1105824 (PMC10188967; doi:10.3389/fpsyg.2023.1105824)
Supplement: Supplementary file 1 [file table_1.docx]

The stimuli’ valance and arousal

The auditory stimuli were created on the basis of pretest. To generate bimodal audio-visual stimuli, each face was randomly paired with a voice of the same category (e.g. a male-threatening face was paired with a randomly selected male-threatening voice), resulting in 30 threatening and 30 non-threatening bimodal face-voice pairs. Stimuli served as the moving targets in the TTC task. In the unimodal visual condition, the moving target was either a threatening or non-threatening face. In the audio-visual condition, the moving target was either a threatening face paired with a threatening voice or a non-threatening face paired with a non-threatening voice. Participants rated audio-visual stimuli’s arousal and valence value on a 1 (low) to 9 (high) Likert scale. Specifically, to rate the valence level, we asked participants to rate how valence each stimulus was. To rate the arousal level, we asked them how arousing each stimulus was (Table 1). For the valance, the smaller the number is, the more significant the threatening/negative emotion, the larger the number is, the more significant the non-threatening/positive emotion, and 5 represents the neutral emotion in between. In terms of arousal, the smaller the number is, the dull the participant is; the larger the number is the more emotional fluctuations the participant can evoke, and the more excitement, enthusiasm, tension, or stimulation they can feel.

In Experiment 1 and 2, we tested thirty participants (age range 17–25, 11 males) to rate the stimuli. Paired sample t-test showed that the arousal of threatening faces (6.93 ± 0.61) was significantly higher than that of non-threatening faces (5.15 ± 0.40), t (29) = 13.22, p < 0.001. The arousal of the audiovisual threatening target (6.99 ± 0.41) was significantly higher than that of the audiovisual non-threatening target (5.77 ± 0.35), t (29) = 14.10, p < 0.001.

In Experiment 3, we tested twenty-three participants (age range 18–24, 5 males) to rate the stimuli. Paired sample t-test showed that the arousal of audiovisual threatening target (7.23± 0.37) was significantly higher than that of audiovisual non-threatening target (6.00 ± 0.35), *t* (29) = 12.31, *p* < 0.001 (Table 1).

**Table 1** stimuli’ valance and arousal rate

| Categories | Visual target | | Audiovisual target in Experimen1 | | Audiovisual target in Experimen3 | |
| --- | --- | --- | --- | --- | --- | --- |
|  | [valence](javascript:;) | [arousal](javascript:;) | [valence](javascript:;) | [arousal](javascript:;) | [valence](javascript:;) | [arousal](javascript:;) |
| Threat | 2.10(±0.44) | 7.15(±0.52) | 2.10(±0.38) | 6.99(±0.41) | 1.85(±0.36) | 7.23(±0.37) |
| Non-threat | 7.00(±0.28) | 5.42(±0.51) | 6.89(±0.31) | 5.77(±0.35) | 6.73(±0.34) | 6.00(±0.35) |
